# SAP BusinessObjects in Medical Informatics

**DOI:** 10.7759/cureus.40208

**Published:** 2023-06-10

**Authors:** Iwan P Sofjan, Irim Salik, Peter J Panzica

**Affiliations:** 1 Anesthesiology, Westchester Medical Center, Valhalla, USA

**Keywords:** multidimensional database (mdb), sap businessobjects ®, medical informatics, anesthesia information management system (aims), electronic health record (ehr)

## Abstract

Electronic health record (EHR) generates a large amount of data filled with opportunities to enhance documentation compliance, quality improvement, and other metrics. Various software tools exist, but many clinicians are unaware of them. Our institution switched from a hybrid of paper and multiple small EHRs to one all-inclusive EHR system. We faced significant challenges beyond the typical new software deployment phase that affected our departmental regulatory compliance, quality measures, and research initiatives. We aimed to navigate these issues through the use of medical informatics. We used a multidimensional database software analysis tool called SAP BusinessObjects® (SAP SE. Released 2020. SAP BusinessObjects, Version 14.2.8.3671. Waldorf, Germany) to design automated queries for the patient database to generate various reports for our department. As a result, We improved our anesthesia documentation non-compliance from 13-17% of all cases to 4% within months. We have also used this tool to automatically generate various reports such as preoperative beta-blocker administrations, caseloads, case complications, procedure logs, and medication records. Even today many departments rely on manual checks for even the most basic documentation and quality metric compliance, which can be time consuming and costly. Using medical informatics tools is a highly efficient alternative. Fortunately, many software tools exist within most modern EHR packages, and most people can learn to use these tools productively.

## Introduction

Healthcare organizations have been incentivized to adopt electronic health record (EHR) systems by a sizeable federal investment following the enactment of the Health Information Technology for Economic and Clinical Health (HITECH) Act in 2009 [1]. The HITECH Act was an important element of the Accountable Care Act, levying financial penalties on healthcare providers who fail to meet basic standards for Medicare and Medicaid patients [2]. As a result, the traditional “fee-for-service” model has been replaced by outcomes-based reimbursement, predicated on empirical clinical evidence of patient benefit [3]. 

Anesthesia Information Management System (AIMS) refers to a software that allows for automatic perioperative data collection and subsequent retrieval. In the field of anesthesiology, these data include the various hemodynamic parameters, medications, procedures, intraoperative events, billing items, and many others. Some AIMS also include various clinical decision support (CDS) tools that generate multiple reminders, notifications, and alerts to increase efficiency and reduce errors throughout anesthesia delivery [4-9]. 

The adoption of AIMS or EHR, in general, has and will continue to grow rapidly. For instance, AIMS adoption increased from only 10% in 2007 to 84% in 2020 across all medical institutions. With this trend, there is a growing demand for healthcare informatics professionals to correlate healthcare delivery and the information sciences [10-14]. This process typically includes a subset of physicians working with various staff from the EHR company. Unfortunately, this process can be very inefficient especially when there are budgetary and time constraints. However, there are several powerful software that clinicians can learn and eventually customize on their own to produce various reports.

## Technical report

Objective

Our institution switched from a hybrid of paper and multiple standalone electronic medical record software to one all-inclusive EHR which includes an AIMS module in 2020. We faced many challenges beyond the typical new software deployment phase that affected our departmental regulatory compliance, quality measures, and research initiatives. We addressed these challenges through the use of informatics. Specifically, we will describe the use of a database analysis software called SAP BusinessObjects® (SAP BO, Version 14.2.8.3671, SAP SE, Waldorf, Germany), which allows for highly customized reports that are otherwise very labor-intensive and time-consuming to generate. We will show examples of its application in improving compliance in perioperative anesthesia documentation, preoperative beta-blocker utilization, tracking caseload, procedures, and complications, as well as the implication for forecasting expenses of practice.

Materials and methods

**Figure 1:**
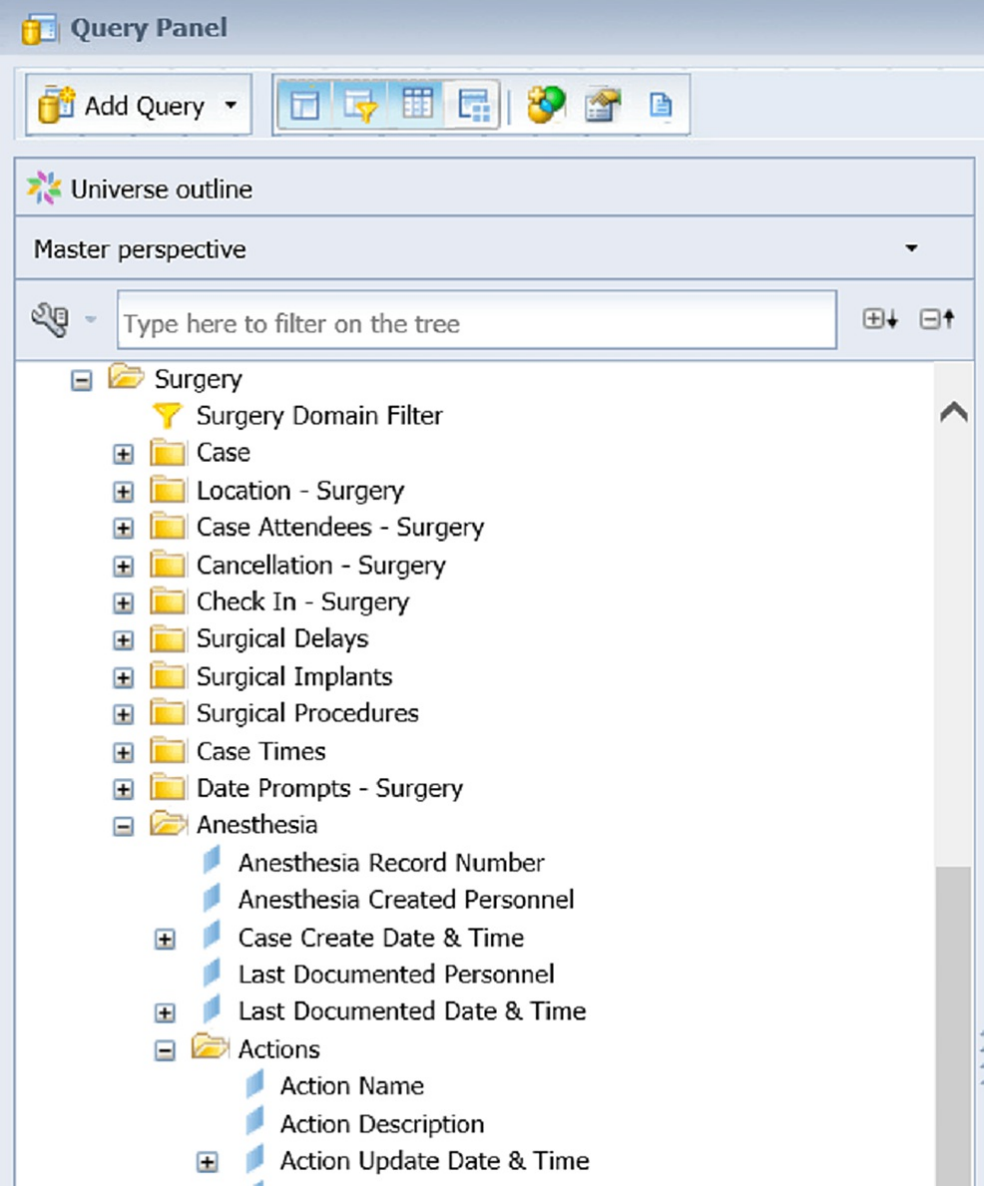
A sample of the Universe

One of our EHR issues after transitioning to AIMS is documenting proper pre-anesthesia and post-anesthesia notes for every case, which is a standard requirement set forth by the Centers for Medicare and Medicaid (CMS). Unfortunately, the AIMS we have cannot automatically check for the presence of the pre-anesthesia and post-anesthesia notes. Often these missing notes are not caught until someone from the medical record office manually checks the charts long after the case was done. To address this, we designed an SAP BO query to generate a list of the relevant electronic documents which include the anesthesia record, pre-anesthesia, and post-anesthesia notes. In SAP BO, the patient data are stored and organized in a multidimensional database (MDB) structure called the universe, as shown in Figure 1.

**Figure 2:**
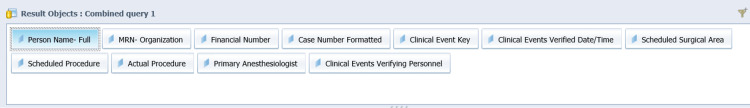
A sample of Result Objects

From the universe, we can select relevant data points that we want to include in our report, as shown in the Result Objects window in Figure 2.

**Figure 3:**
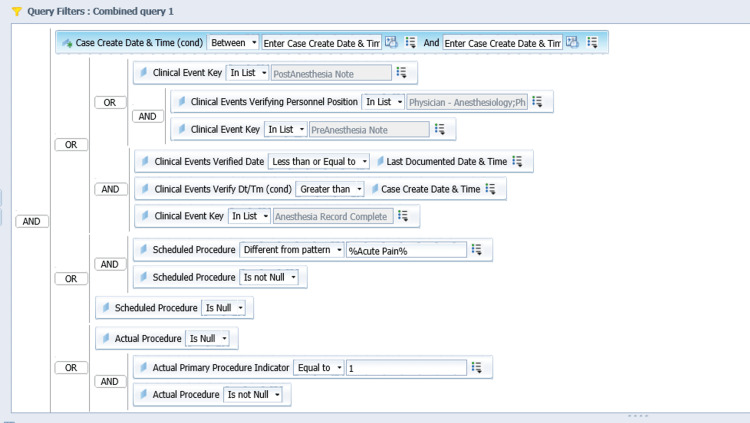
A sample of Query Filters

**Figure 4:**
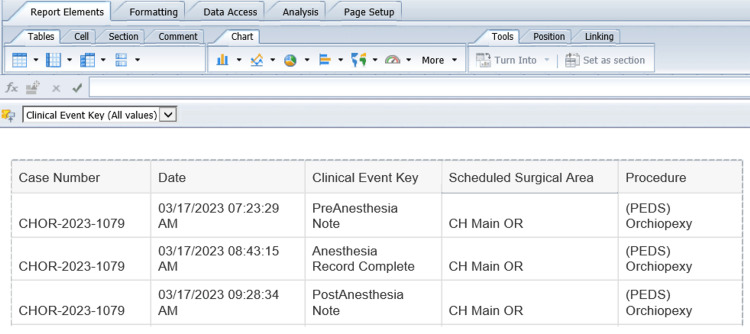
A sample Output

The next step is designing the query to filter for the specific items of interest. In this example query, we are searching for anesthesia records, pre-anesthesia notes, and post-anesthesia notes that meet certain criteria (e.g. date of creation, exclusion of some procedures, verified by an attending anesthesiologist, etc.) as depicted in Figure 3. A sample output from this query is shown in Figure 4. We then used Microsoft Excel® (Version 2211, Microsoft Corporation, Redmond, United States) to analyze the data, in particular, whether each anesthesia record has an associated pre-anesthesia and post-anesthesia notes. We organized the compliance data based on the primary anesthesiology attendings and track the rate on a month-by-month basis.

Results

**Figure 5:**
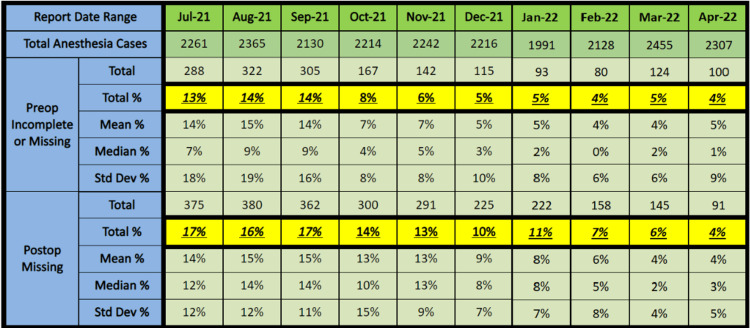
Departmental note compliance rate

Figure 5 shows the compliance improvement we achieved within months. Our initial overall non-compliance rate was 13% for the pre-anesthesia notes and 17% for the post-anesthesia notes. With regular notifications and education, especially for the chronically non-compliant attendings, gradual improvements were seen. By the ninth month, the overall non-compliance rate was reduced to 4%.

**Figure 6:**
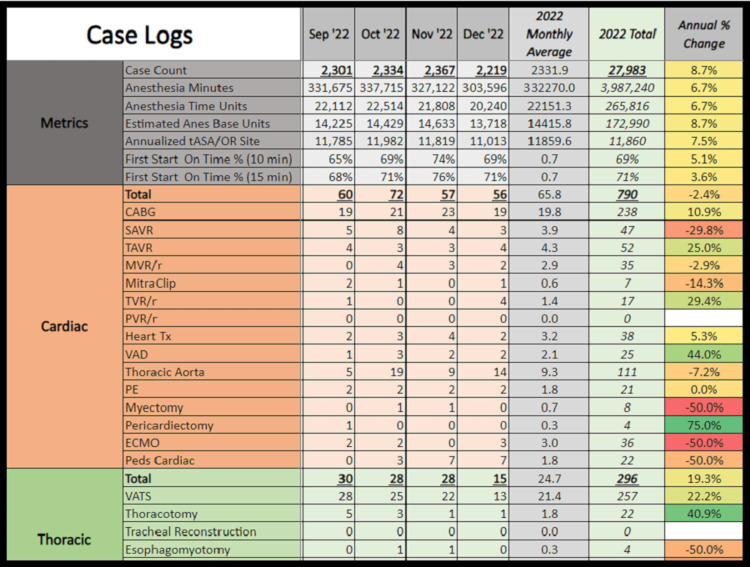
Departmental case logs

**Figure 7:**
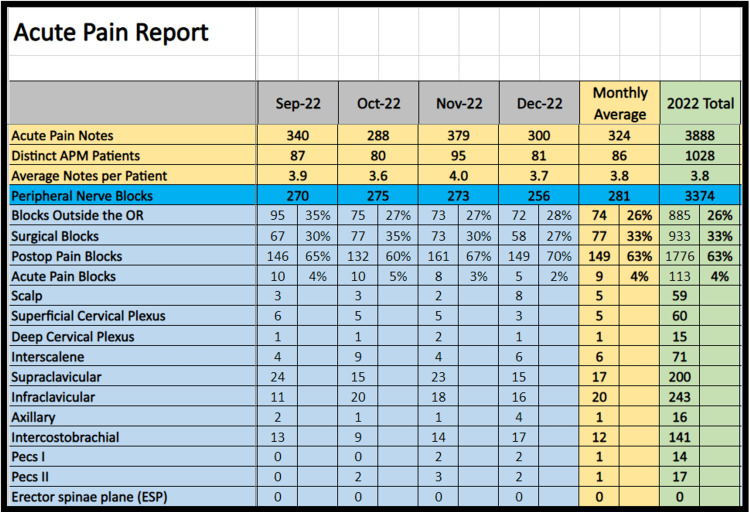
Acute pain medicine patient notes and procedures report

The SAP BO query can be customized in many ways to rapidly peruse through tens of thousands of charts to check items of relevance. For instance, we can now generate monthly reports for our anesthesia caseload, shown in Figure 6, and procedures such as nerve blocks shown in Figure 7. Furthermore, we have also SAP BO to track intraoperative complications, medication usage, staff case logs, case times, and many others. Once designed, these queries can be rerun quickly compared to manually tallying the data points. We have also started using SAP BO to improve research data collection, resident case logs, and billing compliance, among others.

## Discussion

High-level medical informatics to navigate complex EHR systems is pivotal to delivering more efficient care, enabling translational research, optimizing healthcare operational productivity, and reducing cost [15-16]. Unfortunately, easily accessible information technology resources are often lacking in smaller medical systems. The usability of the EHR system is also encumbered by a lack of standardized user interfaces and the lack of trained professionals to navigate the platform. Regulatory agencies and governing bodies should ensure EHR interoperability and reduce reliance on specified vendors. The healthcare industry, as opposed to EHR vendors, should be at the forefront of this mission in order to uphold ethical standards, data privacy, security, and confidentiality. 

While AIMS implementation is costly for an institution, the return on investment is reflected via reduced anesthesia medication expenditure, improved charge and billing capture, increased reimbursement revenue from improved hospital coding, and more efficient staff scheduling [17,18]. Healthcare finance heavily depends on the field of analytics, via 1) cost savings based on improved patient care and outcomes and 2) identification of revenue leakage. Most healthcare organizations detect revenue leakage through analytics review and manual audits, rendering this process time-consuming and error-prone. As an alternative, predictive modeling and machine learning can be automated to recognize erroneous or missing patterns in billing records. This dual method of computer-based analytics, in conjunction with human review from the billing department, has been shown to reduce expenses by 75% in one health system [19]. 

The field of anesthesiology in particular is ripe for the application of "Big Data" to outcomes research, quality improvement, and practice management. There is a myriad of perioperative data available for analysis to improve patient care and clinical efficiency. By learning about the analysis tools available, anesthesia practitioners can play a pivotal role in the future of analytics to implement the innovation necessary for the optimization of our dynamic healthcare ecosystem.

## Conclusions

Electronic health record complexity, usage, and dependence will continue to grow. Unfortunately, for most hospitals, there are limited amounts of IT resources available to help navigate the large amount of data. As medical practitioners, learning how to independently generate customized reports and data analysis can provide a lot of value while saving time and money. Software tools such as SAP BusinessObjects®, used in many major industries, exist and in many instances are built-in as part of the EHR installation package. With self or formal education, most clinicians can learn to utilize these tools to enhance compliance, quality improvement, cost saving, workload tracking, research efforts, and many others.
